# The immediate adverse drug reactions induced by ShenMai Injection are mediated by thymus-derived T cells and associated with RhoA/ROCK signaling pathway

**DOI:** 10.3389/fimmu.2023.1135701

**Published:** 2023-03-21

**Authors:** Shan Jiang, Bo Sun, Yan Zhang, Jiayin Han, Yanyan Zhou, Chen Pan, Hongjie Wang, Nan Si, Baolin Bian, Linna Wang, Lifang Wang, Xiaolu Wei, Haiyu Zhao

**Affiliations:** Institute of Chinese Materia Medica, China Academy of Chinese Medical Sciences, Beijing, China

**Keywords:** Shenmai injection, T cell, RhoA/ROCK signaling pathway, vascular leakage, lysolecithin, cytokine

## Abstract

**Introduction:**

The mechanism of the immediate adverse drug reactions (ADRs) induced by ShenMai injection (SMI) has not been completely elucidated. Within 30 minutes, the ears and lungs of mice injected with SMI for the first time showed edema and exudation reactions. These reactions were different from the IV hypersensitivity. The theory of pharmacological interaction with immune receptor (p-i) offered a new insight into the mechanisms of immediate ADRs induced by SMI.

**Methods:**

In this study, we determined that the ADRs were mediated by thymus-derived T cells through the different reactions of BALB/c mice (thymus-derived T cell normal) and BALB/c nude mice (thymus-derived T cell deficient) after injecting SMI. The flow cytometric analysis, cytokine bead array (CBA) assay and untargeted metabolomics were used to explain the mechanisms of the immediate ADRs. Moreover, the activation of the RhoA/ROCK signaling pathway was detected by western blot analysis.

**Results:**

In BALB/c mice, the vascular leakage and histopathology results showed the occurrence of the immediate ADRs induced by SMI. The flow cytometric analysis revealed that CD4^+^ T cell subsets (Th1/Th2, Th17/Treg) were imbalanced. And the levels of cytokines such as IL-2, IL-4, IL12P70 and INF-γ increased significantly. However, in BALB/c nude mice, all the indicators mentioned above have not changed significantly. The metabolic profile of both BALB/c mice and BALB/c nude mice was significantly changed after injecting SMI, and the notable increase in lysolecithin level might have a greater association with the immediate ADRs induced by SMI. The Spearman correlation analysis revealed that LysoPC (18:3(6Z,9Z,12Z)/0:0) showed a significant positive correlation with cytokines. After injecting SMI, the levels of RhoA/ROCK signaling pathway-related protein increased significantly in BALB/c mice. Protein-protein interaction (PPI) showed that the increased lysolecithin levels might be related to the activation of the RhoA/ROCK signaling pathway.

**Discussion:**

Together, the results of our study revealed that the immediate ADRs induced by SMI were mediated by thymus-derived T cells, and elucidated the mechanisms of such ADRs. This study provided new insights into the underlying mechanism of immediate ADRs induced by SMI.

## Introduction

Natural medicine injections are an important method in the application of natural medicine. It was an innovation that has been proven to have high bioavailability and rapid curative effects in extensive clinical applications (1). ShenMai injection (SMI) is a natural medicine injection product for treating coronary atherosclerotic cardiopathy and viral myocarditis. The main medicinal compositions of SMI are red ginseng and Ophiopogon, which contain a variety of effective ingredients such as polysaccharides, saponin, and organic acid (2). Since 1995, the scope of diseases treated by SMI has gradually expanded due to its effectiveness (3). Up to now, evidence-based advances revealed that SMI exerted potent effects against COVID-19 (4, 5). However, with the widespread application of SMI, its adverse drug reactions (ADRs) such as systemic reactions (allergic shock), skin lesions (urticaria, pruritus), bronchospasm and local pain (3, 6) have become increasingly prominent. Ginsenoside Rb1, ginsenoside Rb2, ginsenoside Rc, ginsenoside Rd and 20 (S)-ginsenoside Rg3 were the main components in SMI that could cause ADRs (7). In 215 (80.90%) of the 246 ADR cases caused by SMI, ADRs occurred the first time when the medication was given (3). Unlike IV hypersensitivity, this type of ADR usually occurred after the first exposure (30 min) to antigen without prior sensitization.

In recent years, cellular immune-mediated ADRs have received increasing attention. Previous theories have stated that small molecular drugs could be combined with endogenous peptides covalently to form antigen-hapten carrier complexes. Then, they were presented to the human leukocyte antigen (HLA) molecule and recognized by T cell receptors (TCRs), thus inducing a drug-specific cellular immune response (8, 9). However, the pharmacological interaction with immune receptor (p-i) concept postulated that small-molecule drugs such as Oxypurinol (10) might directly stimulate the helper T cells and/or cytotoxic T-lymphocyte (CD4^+^ and/or CD8^+^ T cells) (11). This theory offered a new opportunity to understand how immediate ADRs could be initiated by small-molecule components in SMI. At the same time, the advantages of metabolomics in toxicology studies are gradually emerging. Metabolomics is an effective tool for unbiased detection and analysis of the whole set of metabolites to screen out different metabolites (12). The use of untargeted metabolomics technologies to discover and identify differential endogenous metabolites allowed for rapid, accurate studies of ADRs mechanisms.

The Ras homolog family member A (RhoA) and its downstream effector Rho-associated kinase (ROCK) were involved in the process of cytoskeletal rearrangement, reactive oxygen species (ROS) production and morphology (13, 14). What’s more, the RhoA/ROCK signaling pathway could respond to various chemicals or mediators by regulating endothelial permeability (14–16). The clinical signs of immediate ADRs induced by SMI including allergic shock, skin lesions and bronchospasm were closely related to endothelial hyperpermeability. Therefore, we speculated that the activation of the RhoA/ROCK signaling pathway might be related to the occurrence of ADRs induced by SMI.

The present study confirmed that the immediate ADRs caused by SMI might be mediated by thymus-derived T cells using BALB/c mice and BALB/c nude mice. We further explored the disturbed RhoA/ROCK signaling pathway in SMI-induced ADRs using mice models. In addition, this was the first study to investigate the mechanism of immediate ADRs induced by thymus-derived T cells using metabolomics during SMI treatment. Overall, our study presented new insights related to the immediate ADRs induced by SMI and these results provided valuable data to support further research on the role of T-cell immunity in clinical ADRs.

## Materials and methods

### Animals

Thirty male BALB/c mice (SPF level,18-20 g) and thirty male BALB/c nude mice (SPF level, 18-20 g) were purchased from Vital River Laboratory Animal Technology, Co., Ltd. (Beijing, China). The mice were raised (temperature (24 ± 2 °C), humidity (60 ± 5%), 12 h dark/light cycle) in an SPF-level breeding room. All mice were fed the standard purified rodent diet (Research Diets, D10001) and water. After 3 days of acclimation, BALB/c mice were randomized into a control group (BS, injection of saline, 15 mice) and an experimental group (BSMI, injection of SMI, 15 mice) based on the body weight. Similarly, the BALB/c nude mice were divided into a control group (NS, injection of saline, 15 mice) and an experimental group (NSMI, injection of SMI, 15 mice). All animal experiments were performed following the Guide for the Care and Use of Laboratory Animals. The animal protocols were approved by the Institutional Animal Care and Use Committee of the Institute of Chinese Materia Medica, China Academy of Chinese Medical Sciences (approval certificate number: 2022B196.)

### Vascular leakage test after SMI injection

According to the recommended clinical dose of SMI (1.5 mL/kg), mice in the experimental group were given a dose of 30 mL/kg (2 x the clinical dose), which was calculated according to the animal dose conversion equation (FDA, 2005). In this study, the mice were injected with saline or SMI containing 0.4% EB in the tail vein. After 30 min of drug/EB injection, the ears of mice were collected and stored in 2 mL formamide for EB extraction. To assess the vascular leakage, the amount of EB extravasation in the ear was evaluated using a microplate reader at an absorption wavelength of 610 nm (Thermo Scientific Varioskan Flash, Thermo Fisher Scientific, United States).

### Evaluation of histological examination

The lungs of mice were preserved in 10% neutral-buffered formalin for further staining. After dehydration with gradient alcohol, transparent with xylene, the tissue was embedded in paraffin. 5 μm thick sections were cut for hematoxylin and eosin (H&E) staining. The stained samples of the lung tissues were observed and imaged using a light microscope (DM1000, Leica Microsystems, Wetzlar, Germany).

### Cytokine detection in lung

A flowcytometric cytokine bead array (CBA) assay has now been used to determine multiple interleukins, simultaneously. This study determined the cytokine (IL-2, IL-4, IL-6, IL-12p70 and INF-γ) profiles of interleukins in the lung of mice by using multiplex Flowcytometric CBA array assay. According to the literature, the left lung lobe of mice was used to prepare single-cell suspension for CBA array assay (17). Then, the assay was performed using BD™ CBA Flex Set (BD Biosciences, San Jose, CA, USA) according to the manufacturer’s instructions. The data was acquired on a CytoFlex flow cytometer (Beckman Coulter, USA). And the data analyses for all interleukins were performed using FCAP Array Software v3.0 (BD Biosciences, San Jose, CA, USA).

### Flow cytometric analysis of CD4^+^ T cell subsets

According to the literature, the spleen of the mice was used to prepare single-cell suspension (18). Then, the effects of SMI on mice T helper (Th1, Th2, Th17) cells and regulatory T (Treg) cells were determined using flow cytometry. In brief, the spleen of mice was digested and single-cell suspensions were stimulated with 50 ng/mL PMA and 1 μg/mL ionomycin in the presence of BD GolgiPlug (Leuko Act Cktl with GolgiPlug, BD Biosciences, San Jose, CA, USA) for 10 hours. Cells were fixed and permeabilized using BD Transcription Factor Buffer Set (BD Biosciences, San Jose, CA, USA) followed by staining with fluorescence-labeled antibodies CD3e-BV605 (145-2C11), CD4-APC-H7 (GK1.5), CD8a-BV510 (53-6.7), CD25-BV421 (PC61), FoxP3-Alexa 647 (MF23), IFN -γ-FITC (XMG1.2), IL-4-PE-Cy7 (11B11) and IL-17A-PE (TC11-18H10). All fluorescence-labeled monoclonal antibodies were purchased from BD Pharmingen. Flow cytometry was performed on a Beckman Coulter CytoFlex flow cytometry system.

## Untargeted metabolomics analysis of mice plasma

### Sample preparation

As previously described (19), 50 μL plasma was obtained and put into a 0.5 mL centrifuge tube containing 200 μL cold acetonitrile. Then the samples were vortexed (3 min) and centrifuged (12000 rpm, 4 °C, 15 min) in turn. 200 µL supernatant was collected and dried under N_2_ at room temperature. Before further analysis, these residues of samples were re-dissolved using 200 µL of 5% acetonitrile (containing 0.1% formic acid). Exactly 10 μL solution from each sample was taken and mixed into the QC sample for testing the stability of the instrument. The QC sample was inserted in an interval of 5-10 test samples. All reagents used were chromatographically pure.

### Data acquisition for untargeted metabolomics

Metabolic profiling of the mice’s lung tissue was performed with established methods (19). The UHPLC system (Ultimate 3000, Thermo Fisher Scientific, USA) with a UPLC HSS T3 column (2.1 mm×100 mm, 1.8 μm, Waters) was used to perform chromatographic separation at 35°C. Then, the LTQ Orbitrap Velos Pro (Thermo Fisher Scientific, USA) was used to collect the primary and secondary mass spectrometric data. The mobile phase of the UHPLC system was composed of (A) 0.1% formic acid in water and (B) acetonitrile, 95%-45% A; 2.00-10.00 min, 45-5% A; 10.00-15.00 min, 5-5% A; 15.00-15.50 min, 5-95% A; 15.50-20.00 min, 95% A. The flow rate was 0.3 mL/min and the injection volume was 5 µL.

The LTQ Orbitrap Velos Pro was combined with UHPLC *via* an ESI interface. The acquisition software (Xcalibur 3.0, Thermo) continuously evaluated the full scan survey MS data as it collected and triggered the acquisition of MS/MS spectra depending on preselected criteria. The ion spray voltages were set at 3.5 kV in the positive ion mode. The auxiliary gas flow of 10 psi, sheath gas flow of 30 arb, auxiliary gas heater temperature of 350°C, capillary temperature of 320°C, the full mass resolution as 30000 and the MS^2^ experiments were set as data-dependent scans.

### Multivariate analysis of LC-MS/MS data

All the data were processed with the Progenesis QI software (Waters, USA) for imputing raw data, eliminating noise, correcting baseline, aligning and selecting peaks to obtain necessary information, including retention time (tR) and m/z data pairs et al. Next, the acquired data were imported to the SIMCA-P 14.0 software (Umetrics, Sweden) for further analysis. Principal component analysis (PCA) and orthogonal to partial least squares-discriminate analysis (OPLS-DA) were performed to determine whether the metabolic phenotypes were different among groups. Then, the differential metabolites of SMI treated were screened using the judgment methods such as VIP≥1.5, FC≥2 and *p*<0.05. The identification of differential metabolites was performed using an online human metabolome database (https://hmdb.ca/) in Progenesis QI software.

### Spearman correlation analysis

The correlation between cytokines and metabolites was analyzed by the spearman correlation coefficient (https://www.bioincloud.tech/). Correlations with *p < 0.05* were considered significant and the heat maps were used to display the correlations determined. What’s more, a network was drawn to reveal the mechanism of thymus-derived T cell-mediated immediate ADRs in BALB/c mice after injecting SMI.

### Protein-protein interaction network construction

To excavate interactions among metabolites and Rho-associated kinase-dependent pathways, a protein-protein interaction (PPI) network was established using the String database (https://www.string-db.org), with the minimum required interaction score set at 0.950. The metabolites targets were predicted on the Swiss TargetPrediction database (http://www.swisstargetprediction.ch/). Then, the Cytoscape software was applied to modify images downloaded from the String database, and an MCODE plug-in was used to identify important interacted protein-coding genes.

### Western blot analysis

For western blot analysis, the total protein was isolated from the lung tissue using RIPA lysis buffer (65 mM Tris-HCl pH 7.5, 150 mM NaCl, 1 mM EDTA, 1 mM DTT, 1% Nonidet P-40, 0.5% sodium deoxycholate, 0.1% SDS, protease inhibitor cocktail tablets and phosphatase inhibitor tablets) and quantified. Then, the samples were separated by SDS polyacrylamide gel electrophoresis (SDS-PAGE, 8%-12%) and transferred onto polyvinylidene fluoride (PVDF) membranes. The membranes were incubated with respective primary antibodies of anti-p-MLC2 (1:500), anti-MLC2 (1:500), anti-p-MYPT1 (1:500), anti-MYPT1 (1:500), and anti-RhoA (1:500) at 4 °C over-night, followed by incubation with the species-specific HRP-conjugated secondary antibodies (15, 16). Protein bands were detected by enhanced chemiluminescence (Santa Cruz Biotechnology, Santa Cruz, CA, USA). Images of blots were analyzed by Image J software. Reagents information was shown in the **Supporting Information**.

### Statistical analysis

Statistical product and service solutions (SPSS) 22.0 was employed for statistical analysis. The results were presented as Mean ± SD. Data analysis and the intergroup comparison were analyzed by One-way ANOVA and Student’s *t*-test, respectively. *p*< 0.05 indicated a statistically significant difference. The figures were performed using GraphPad Prism 8.0 software.

## Results

### SMI induced immediate ADRs in BALB/c mice

**Figure 1:**
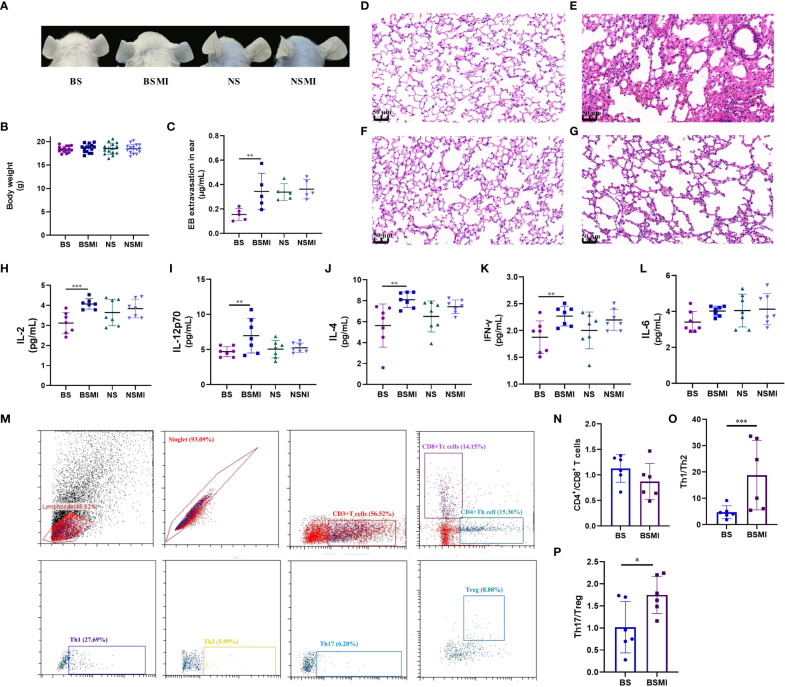
The immediate ADRs induced by SMI may be thymus-derived T cell-mediated in mice. **(A)** Vascular leakage in the ears of BS, BSMI, NS, NSMI group mice after injecting SMI SMI/EB. **(B)** The body weight of mice in BS, BSMI, NS, NSMI groups. **(C)** The results of EB extravasation in BS, BSMI, NS, NSMI groups (*n* = 5 per group). **(D–G)** The result of the microscopic examination in mice lungs (**D**, BS group; **E**, BSMI group; **F**, NS group; **G**, NSMI group; × 200 magnification). **(H–L)** The results of cytokine detection in lungs of mice (H, IL-2; I, IL-12p70; J, IL-4; K, IFN-γ; L, IL-6). **(M)** Flow cytometry figures of CD4^+^ T cell, CD8^+^ T cell, Th1, Th2, Th17 and Treg cell in the spleen. **(N-P)** The results of CD4^+^ T cell subsets (**N**, CD4^+^/CD8^+^ T cell; **O**, Th1/Th2; **P**, Th17/Treg). Data are expressed as Mean ± SD; ∗*p* < 0.05, ∗∗*p* < 0.01, ∗∗∗*p* < 0.001.

To examine the extent of SMI-induced vascular leakage, we used Evans blue (EB) as a marker of plasma protein extravasation. Because of its high affinity for plasma albumin, it could readily form albumin-EB complexes. This property has been widely applied to assess vascular leakage and the extent of inflammatory extravasation (20). We injected SMI intravenously with EB and determined vascular leakage by observing EB leakage in the ear. As a control, EB alone did not cause visible vascular leakage in mice (**Figure 1A**). **Figure 1B** showed the body weight of mice. As shown in **Figure 1C**, EB extravasation in the ear of BALB/c mice occurred within 30 min after the first injection of SMI.

The ADRs induced by SMI were mainly seen in the skin and respiratory system. Therefore, H&E staining was performed on lung tissues to verify the occurrence of ADRs. To evaluate whether SMI caused edema and exudation in BALB/c mice, histological observations (**Figures 1D, E**) of the lungs were obtained at 30 minutes after SMI treatment. Consistent with increased vascular leakage, the microscopic examination confirmed that a single dose of SMI at 30 mL/kg resulted in pulmonary inflammation in BALB/c mice. Besides, BALB/c mice have histological changes such as congestion and edema in the lungs after injecting SMI.

### The immediate ADRs induced by SMI might be thymus-derived T cell-mediated in BALB/c mice

As we all know, the main difference between mice and nude mice is that nude mice have no thymus. The nude mice have been described as primary immunodeficiency mice without mature thymus-derived T cells (21, 22). To demonstrate that the immediate ADRs might be mediated by thymus-derived T cells, we injected both BALB/c nude and BALB/c mice with SMI. In the NSMI group, there was no significant EB leakage in the ear of mice (**Figure 1A**). At the same time, the results of histological observations also showed no signs of lung edema in BALB/c nude mice (**Figures 1F, G**).

Cytokines were thought to play a role in acute and/or immune-mediated ADRs. Cytokines could also be used as biomarkers for predicting adverse events (23). **Figures 1H–L** showed the results of cytokine detection in the lungs of mice. The injection of SMI induced considerable abnormalities in the cytokines of BALB/c mice such as the marked increase in levels of IL-2, IL-12p70, IL-4 and INF-γ (*p*<0.05). However, the levels of IL-2, IL-12p70, IL-4, INF-γ and IL-6 were not significantly increased in the NSMI group compared to the NS group (*p>*0.05). These results suggested that the immediate ADRs appeared to be stronger in BALB/c mice than in BALB/c nude mice.

Furthermore, we used flow cytometry to determine the effect of SMI injection on the T-cell subsets (**Figure 1M**). The CD4^+^/CD8^+^ T cell value of BALB/c mice did not change significantly after SMI injection (*p>*0.05, **Figure 1N**). But the injection of SMI induced the imbalance of Th1/Th2 and Treg/Th17 values in BALB/c mice, as shown in **Figures 1O, P**.

In summary, the immediate ADRs induced by SMI might be thymus-derived T cell-mediated in BALB/c mice.

### The abnormal lysolecithin metabolism might be causally linked to the thymus-derived T cell-mediated immediate ADRs in BALB/c mice after injecting SMI

**Figure 2:**
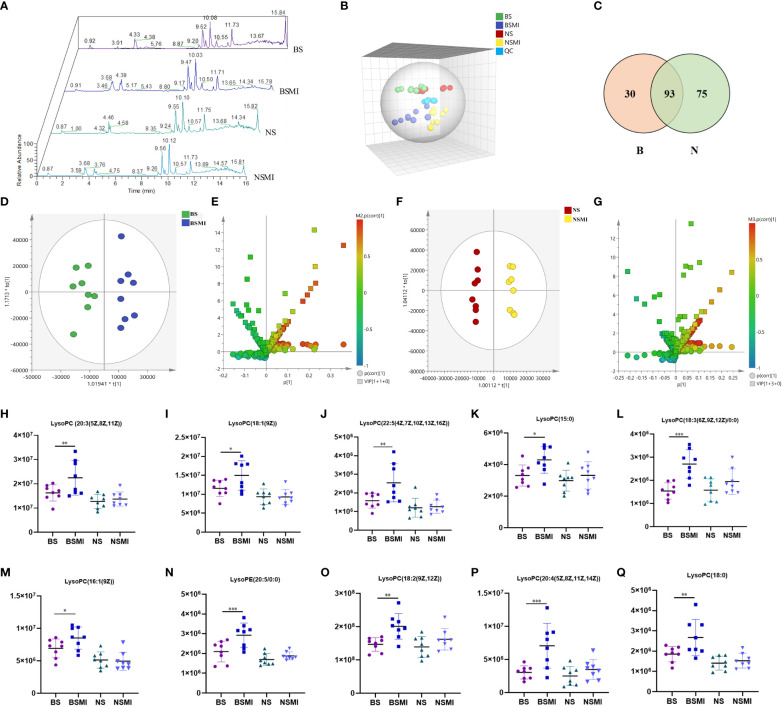
Lysolecithin metabolism may be related to the thymus-derived T cell-mediated immediate ADRs in mice after injecting SMI. **(A)** Total ion chromatogram of mice in BS, BSMI, NS, NSMI groups. **(B)** PCA score plot between the detected samples and QC samples. **(C)** VENN diagram of significantly changed metabolites after SMI injection in BALB/c mice (B, red) and BALB/c nude mice (N, green). **(D)** OPLS-DA plot of BS and BSMI group. **(E)** S+V-plot of BS and BSMI group. **(F)** OPLS-DA plot of NS and NSMI group. **(G)** S+V-plot of NS and NSMI gruop. **(H–Q)** Results of 10 identified metabolites between BS and BSMI groups. The X-axis represents the group and the Y-axis represents the peak relative intensity of the mass spectrum. Data are expressed as Mean ± SD; ∗*p <* 0.05, ∗∗*p <* 0.01, ∗∗∗*p <* 0.001.

Under the optimal UPLC-ESI-Orbitrap MS conditions, metabolites could get a better separation and effective response effect in positive scan mode, and the total ion chromatograms were shown in **Figure 2A**. From the untargeted metabolomics analyses, 1812 metabolites were detected in the plasma samples. Many metabolites were observed to be statistically significant (*p ≤* 0.05, fold change≥1.2 and VIP >1) in numerous pairwise comparisons related to the with or without SMI treated (BS *vs* BSMI, NS *vs* NSMI). A global view of the multi-dimensional data illustrated that plasma differences were observed between the different groups of mice. PCA, displayed in **Figure 2B**, highlighted global changes between saline and SMI injections in mice. The QC samples clustered together displayed satisfactory data quality. Notably, the overlap was observed between saline-injected BALB/c nude and BALB/c mice, highlighting the small metabolic differences between the two groups.

For OPLS-DA analysis (**Figures 2D, F**), the intergroup separation could be apparently observed between the BS and the BSMI group, as well as the NS group and the NSMI group. The parameters of the OPLS-DA score plot obtained from BS and BSMI groups were *R^2^X* = 0.462, *R^2^Y* = 0.914, *Q^2 =^* 0.812 and the *P* value of CV-ANOVA =0.00056. In the NS group and NSMI group, the parameters of the OPLS-DA score plot were *R^2^X* = 0.628, *R^2^Y* = 0.992, *Q^2 =^* 0.822 and the *P* value of CV-ANOVA =0.016. These results suggested OPLS-DA models were robust. The S+V - plot scores (**Figures 2E, G**) were usually used to screen differential metabolites. After the screen by the conditions of VIP >1, fold change (FC) ≥ 1.2 and *p ≤* 0.05, a total of 123 significantly differential metabolites were identified between the BS and BSMI groups. And 168 metabolites were significant changes between the NS and NSMI groups. Among them, 93 metabolites were significantly changed after injection of SMI in both BALB/c nude and BALB/c mice. 30 metabolites showed significant changes only after SMI injection in BALB/c mice, and it was speculated that these 30 metabolites might be related to thymus-derived T cell-mediated immediate ADRs (**Figure 2C**). 10 of the 30 metabolites were identified, including 9 LysoPCs and 1 LysoPE (**Table 1**). Compared to the BS group, injection of SMI induced considerable abnormalities in LysoPC metabolites in the BSMI group, such as significantly increased levels of LysoPC (20:3(5Z,8Z,11Z)), LysoPC(18:1(9Z)), LysoPC(22:5(4Z,7Z,10Z,13Z,16Z)) and et al. in the plasma (*p ≤* 0.05) (**Figures 2H-Q**). However, the levels of the above metabolites were not significantly increased in the NSMI group compared to the NS group (*p>*0.05). These results indicated that the metabolic profiles of mice were significantly altered as a result of SMI injection. Furthermore, abnormal LysoPC metabolism might be associated with the thymus-derived T cell-mediated immediate ADRs of SMI.

**Table 1:** Ten unique metabolites identified in BALB/c mice after injection of SMI.

Num	t_R_ (min)	Ion mode	Formula	Theoretical Mass *m/z*	Experimental Mass *m/z*	Error (ppm)	P value	Max fold Change value	VIP value	Identification
M1	10.14	[M+H] ^+^	C_28_H_53_O_7_NP	546.35541	546.35522	-0.359	3.4E-02	1.3	3.6	LysoPC (20:3(5Z,8Z,11Z))
M2	10.26	[M+H] ^+^	C_26_H_53_O_7_NP	522.35541	522.35547	0.104	3.4E-02	1.3	2.3	LysoPC (18:1(9Z))
M3	10.38	[M+H] ^+^	C_30_H_53_O_7_NP	570.35541	570.35583	0.726	1.8E-02	1.6	1.4	LysoPC (22:5(4Z,7Z,10Z,13Z,16Z))
M4	11.66	[M+H] ^+^	C_23_H_49_O_7_NP	482.32411	482.32422	0.216	6.3E-04	1.4	1.5	LysoPC (15:0)
M5	9.50	[M+H] ^+^	C_26_H_51_O_7_NP	520.33976	520.33990	0.258	2.3E-04	1.4	12.5	LysoPC (18:2(9Z,12Z))
M6	8.85	[M+H] ^+^	C_24_H_49_O_7_NP	494.32411	494.32404	-0.153	2.8E-02	1.3	1.6	LysoPC (16:1(9Z))
M7	9.43	[M+H] ^+^	C_25_H_43_O_7_NP	500.27716	500.27481	-4.709	1.6E-03	1.5	1.1	LysoPE (20:5/0:0)
M8	8.72	[M+H] ^+^	C_26_H_49_O_7_NP	518.32411	518.32404	-0.076	1.1E-05	1.8	1.4	LysoPC (18:3(6Z,9Z,12Z)/0:0)
M9	9.49	[2M+H] ^+^	C_56_H_101_O_14_N_2_P_2_	1087.67225	1087.67017	-1.917	1.3E-03	2.4	3.3	LysoPC (20:4(5Z,8Z,11Z,14Z))
M10	9.89	[M+Na] ^+^	C_26_H_54_NO_7_P	546.35541	546.35577	0.648	1.4E-02	1.5	1.2	LysoPC (18:0)

t_R_,: Retention time.

### The correlation between cytokine and metabolites

**Figure 3:**
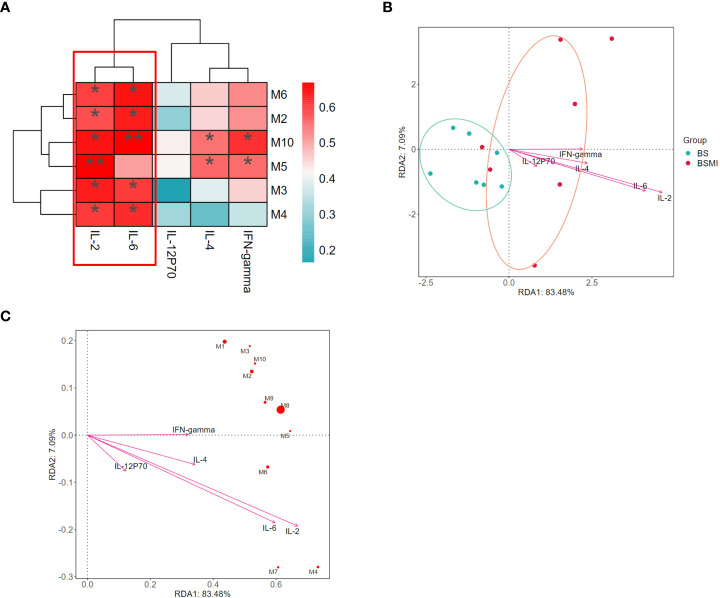
Correlation analysis between the cytokine and metabolites. **(A)** The correlations between lung cytokine and lysolecithin metabolites. **(B)** Db-RDA analysis in BS and BMI group. **(C)** Db-RDA analysis of metabolites and cytokine. ∗*p* < 0.05, ∗∗*p* < 0.01.

The Spearman correlation analysis was used to determine the correlation between metabolic perturbations and cytokine. In **Figure 3A**, multiple metabolites such as LysoPC (18:1(9Z)), LysoPC (22:5(4Z,7Z,10Z,13Z,16Z)), LysoPC (15:0), LysoPC (16:1(9Z)), LysoPC (18:0) showed significantly positive correlations with the levels of IL-2 and IL-6 (p<0.05).

At the same time, the results of distance-based redundancy analysis (db-RDA) also showed that IL-2 and IL-6 were most correlated with the metabolites (**Figures 3B, C**). In addition, compared to the other metabolites, LysoPC (18:3(6Z,9Z,12Z)/0:0) (*p = 0.008*) had the strongest correlation with cytokines. Thus, LysoPC (18:3(6Z,9Z,12Z)/0:0) might be a potential metabolite marker for the immediate ADRs induced by SMI.

### The RhoA/ROCK signaling pathway was activated in the thymus-derived T cell-mediated immediate ADRs of SMI

**Figure 4:**
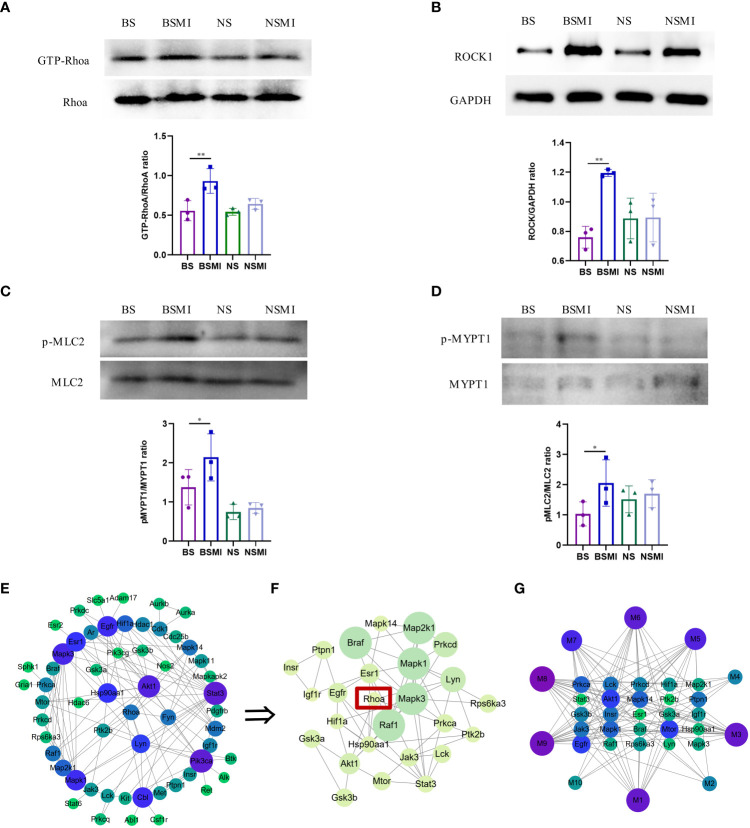
RhoA/ROCK signaling pathway was activated in mice. **(A–D)** Protein validation results of ROCK1/GAPDH, p-MLC2/MLC2, GTP-RhoA/RhoA, p-MYPT1/MYPT1 in the lungs of mice (n = 3, per group). ∗*p* < 0.05, ∗∗*p* < 0.01. **(E)** PPI network of RhoA and protein-coding genes of the 10 lysolecithin targets. The node size is positively correlated with the degree. The darker color indicated stronger correlations. **(F)** The significant module in the PPI containing 26 genes is visualized using the MCODE plug-in. The node size is positively correlated with the MCODE score. The darker color indicated stronger correlations. **(G)** The interactions of the 10 lysolecithin metabolites and the 25 protein-coding genes. The node size is positively correlated with the degree. The darker color indicated stronger correlations.

Previous studies have revealed that the activation of RhoA/ROCK signaling pathways was associated with elevated levels of vascular leakage (15, 16). To verify whether this pathway was associated with the thymus-derived T cell-mediated immediate ADRs, the western blot analysis was conducted in this study. As shown in **Figure 4**, the protein levels of GTP-bound RhoA (GTP-RhoA) (**Figure 4A**), ROCK1(**Figure 4B**), phospho-myosin light chain 2 (p-MLC2) (**Figure 4C**), and phospho-myosin phosphatase targeting subunit 1 (p-MYPT1) (**Figure 4D**) in BALB/c mice lungs were significantly increased after injecting SMI (*p* < 0.05), while the increased levels of the above proteins in BALB/c nude mice lungs were not significant (*p* > 0.05). These results indicated that the increased vascular leakage in mice with ADRs was related to the activation of the RhoA/ROCK signaling pathway.

The increased LysoPCs levels could induce considerable inflammatory reactivity mediated by rho kinase-dependent pathways (24). In order to determine whether there was an interaction relationship between RhoA and lysolecithin, PPI networks were established. The targets of 9 LysoPCs and 1 LysoPE were shown in the **Supporting Information, Table S1**. As shown in **Figure 4E**, the 57 genes could interact with each other. A most active module containing 25 genes activated by 10 lysolecithin metabolites was visualized by the MCODE plug-in (**Figure 4F**). And the interaction network of the 10 metabolites and the 25 genes was shown in **Figure 4G**.

## Discussion

Natural medicine injections have been widely used in clinics, while adverse reaction reports also have increased rapidly in recent years. A study reported that SMI ranked in the top 5 for the incidence of ADRs (25). As natural medicine injections were usually prepared from extracts of a variety of herbs, they contained a complex chemical composition. Supported by the traditional Chinese medical science theory of “multiple ingredients and multiple targets”, most of the chemical components of herbs, such as amino acids and organic acids, were retained in the preparation process. In addition, pharmaceutical adjuvants such as polysorbate 80 and mannitol might also be present in the formulation of natural medicine injections. Thus, multiple classes of components with different chemical properties created great difficulties in the quality control of natural medicine injections and increased the risk of ADRs. In addition, limited studies revealed that the ADRs induced by SMI were usually related to injection speed, solvent type et al. and were dose-dependent (26–28). Therefore, standardizing the application of natural medicine injections and strengthening the observation of patients within 30 minutes of medication could effectively alleviate the immediate ADRs induced by natural medicine injections.

In this study, the dose was approximately 2 times as high as the clinical value and SMI was administered by one-time tail vein injection, which could cause immediate ADRs within 30 min. The symptoms associated with SMI-induced immediate ADRs mainly involved allergic shock, skin lesions and bronchospasm (3, 6). All of these symptoms were associated with the enhancement of vascular permeability. The occurrence of immediate ADRs has limited the clinical use of SMI. Our study explored the mechanism of SMI-induced immediate ADRs using the mice model, which could provide a reference for the prevention of ADRs caused by the clinical use of natural drug injections. In this study, we found significantly increased levels of EB extravasation in the BSMI group within 30 min after injecting SMI. However, there was no significant difference in EB extravasation level between the NS group and the NSMI group. Meanwhile, the histological observation of lung tissue was consistent with the results of the vascular leakage test. The BALB/c mice showed obvious pulmonary edema after injecting SMI, while the nude mice did not. Therefore, the immediate ADRs induced by SMI might be mediated by thymus-derived T cells probably. Type IV hypersensitivity mediated by T cells, also known as delayed-type reactions, usually occurs more than 12 hours after exposure to the anaphylactogen (29). The BALB/c mice that have been continuously fed the standard purified rodent diet appeared ADRS after the first exposure (30 min) to SMI. Thus, the immediate ADRs induced by SMI were distinct from type IV hypersensitivity reactions.

T cell-mediated ADRs involved interactions between small-molecule drugs, HLAs and TCRs, as was the case with most ADRs (30). Currently, three main models for T-cell-mediated ADRs were discussed: the hapten/prohapten model, the pharmacological interactions of drugs with immune receptors (p-i) concept and the altered peptide repertoire model (31). Among them, the p-i concept proposed by Pichler (32) stated that causative drugs bonded non-covalently to HLAs and/or TCRs and directly stimulated specific TCRs to produce drug-reactive T cells. This reaction could usually occur immediately. Specific HLA molecules might have a higher binding affinity for specific drug antigens. It could present drug antigens to specific TCRs, causing a series of T cell activation responses and adverse immune reactions (31, 33). The literature reported that both CD4^+^ and CD8^+^ T cells seemed to participate in the pathogenesis of drug-induced ADRs but played distinct roles. CD4^+^ T cells were first activated and initiated a drug-induced immune response. CD8^+^ T cells then recognized viral antigens and initiated an antiviral immune response, thereby targeting multiple organs and amplifying the inflammatory response (30, 34). Whilst the ELISpot assay was highly sensitive for the detection of drug-responsive T cells, flow cytometry techniques have been critical in the characterization of T-cell phenotypes (35). In this study, the result of flow cytometric analysis showed the proportion of CD4^+^/CD8^+^ T cells increased, but it did not reach a significant level. Moreover, as compared with control mice, the CD4^+^ T cell subsets were influenced in BALB/c mice after injecting SMI, such as the imbalance of Th1/Th2 and Th17/Treg levels. Obviously, the imbalance of the T cell subsets could not be caused by cellular proliferation in a short period of time. T cells made timely contact with antigen-bearing cells through targeted migration (homing) and T cell trafficking to provide immune protection (36). At the same time, T cell subsets could be selectively recruited and local inflammation accelerated the rate at which T cells entered the lymph nodes (37, 38). Therefore, we thought that the imbalance of the T cell subsets after injecting SMI might be related to T cell trafficking and the selective recruitment of the T cell subsets (39). To a certain extent, these results indicated that thymus-derived T cells participated in the immediate ADRs induced by SMI.

T cells underwent a process known as clonal expansion upon activation. The cytokines released during this process were thought to play a role in acute and/or immune-mediated ADRs for downstream reaction activation (23, 40). Th1 cells contributed to cell-mediated inflammatory immunity, while Th2 cells were responsible for humoral responses. The Th1 cytokines IFN-γ and IL-2 have been identified as key diagnostic factors indicating the involvement of drug-specific T cells in ADRs (41–43). The results of our study suggested that BALB/c mice injected with SMI had significantly higher Th1/Th2 levels, as well as IFN-γ and IL-2 levels, compared to BALB/c mice injected with normal saline. As a pro-inflammatory cytokine that induced the differentiation of CD4^+^ T cells into Th1 cells, the levels of IL-12 p70 were also significantly increased in BALB/c mice injected with SMI. Similarly, IL4 and IL6 levels also showed increasing trends. However, the levels of IFN-γ, IL-2, IL-12 p70, IL4 and IL6 were not significantly increased in the NSMI group mice compared to the NS group mice. The results presented here demonstrated that thymus-derived T cells played an essential role in SMI-induced ADRs.

The metabolite profiles were studied in detail by metabolomics to explore the mechanisms of thymus-derived T cell-mediated ADRs after injecting SMI (44). The current study found SMI significantly altered the metabolic profile of BALB/c mice and BALB/c nude mice *in vivo*. In the results of the Venn analysis, more than 90 metabolites of BALB/c mice and BALB/c nude were simultaneously altered, which might be mostly related to the positive effects of SMI. There were 10 identified metabolites with significant changes only in BALB/c mice after injecting SMI: 9 for LysoPCs and 1 for LysoPE. Thus, it was speculated that these 10 metabolites might be related to thymus-derived T cell-mediated immediate ADRs after injecting SMI. Previous reports indicated that LysoPCs had immunomodulatory functions, as they induced the expression of multiple genes associated with inflammation in endothelial cells, smooth muscle cells, macrophages and T cells, including cytokines and chemokines, adhesion molecules, growth factors and pro-inflammatory enzymes (45–47). Coincidentally, the result of the correlation between cytokine and metabolites showed that six LysoPCs were positively correlated with IL2, IL4, IL6 and INF-γ. Among them, LysoPC (18:2(9Z,12Z)) was more closely related to the above four cytokines. These results suggested the potential of LysoPC (18:2(9Z,12Z)) as a marker of immediate ADRs induced by SMI.

Many reports have argued that LysoPCs induced considerable neuroinflammatory reactivity in glia mediated by Rho-associated kinase-dependent pathways. Moreover, LysoPCs could activate rho kinase signaling in various cell types such as endothelial cells and smooth muscle cells (24). The PPI network showed that RhoA interacted with the lysolecithin metabolites targets, indicating that the increased level of the 10 lysolecithin metabolites might be related to the activation of the RhoA/ROCK signaling pathway. RhoA could regulate vascular endothelial permeability, which was correlated with vascular leakage. When RhoA formed an active GTP-bound state, it activated the downstream effector ROCK.

Then, the activation of ROCK promoted the phosphorylation of myosin light chain (MLC) and myosin phosphatase targeting subunit 1 (MYPT1), which could aggravate endothelial dysfunction and inflammation (48, 49). Our results demonstrated the RhoA/ROCK signaling pathway was significantly activated in BALB/c mice after injecting SMI, which corresponded to significant increases in vascular leakage and LysoPCs levels. Overall, significant increases in levels of lysolecithin metabolites and RhoA/ROCK signaling pathway-related proteins were only observed in BALB/c mice after simultaneous injecting SMI in BALB/c mice (thymus-derived T cells normal) and BALB/c nude mice (thymus-derived T cells deficient). These results revealed that the presence and absence of thymus-derived T cells were associated with the severity of immediate ADRs induced by SMI as well as the levels of lysolecithin metabolites and RhoA/ROCK pathway-related proteins. At the same time, BALB/c nude mice showed non-significant increases in lysolecithin metabolites and RhoA/ROCK pathway-related protein levels. These results suggested that the activation of the RhoA/ROCK pathway by elevated lysolecithin levels might be one of the main mechanisms underlying thymus-derived T cell-mediated immediate ADRs. A previous study showed that Shuanghuanglian injection was able to induce a pseudo-allergic reaction, a non-immune-mediated hypersensitivity reaction, by activating the RhoA/ROCK signaling pathway (15). Our findings confirmed that the RhoA/ROCK signaling pathway could still be activated in immune-mediated ADRs. There are still some deficiencies in this study. Firstly, we have only focused on the effect of SMI-induced immediate ADRs on CD4^+^ T cell subsets in this research. In view of the fact that CD8^+^ T cell plays an important role in immunology, CD8^+^ T cell subsets will be investigated in subsequent studies. The p-i theory claimed that small-molecule components could directly activate T cells by binding to HLA or TCR. The lectin in plants, involved in the lectin pathway of complement activation, could specifically stimulate CD4^+^ T cells (50, 51). In addition, the superantigen could also stimulate powerful T-cell responses *in vivo* (52). Thus, the specific mechanism by which SMI activated T cells needed further study. Eliminate the above deficiencies, all results of this study could still provide supportive data for continued research on immediate ADRs reduced by SMI.

**Figure 5:**
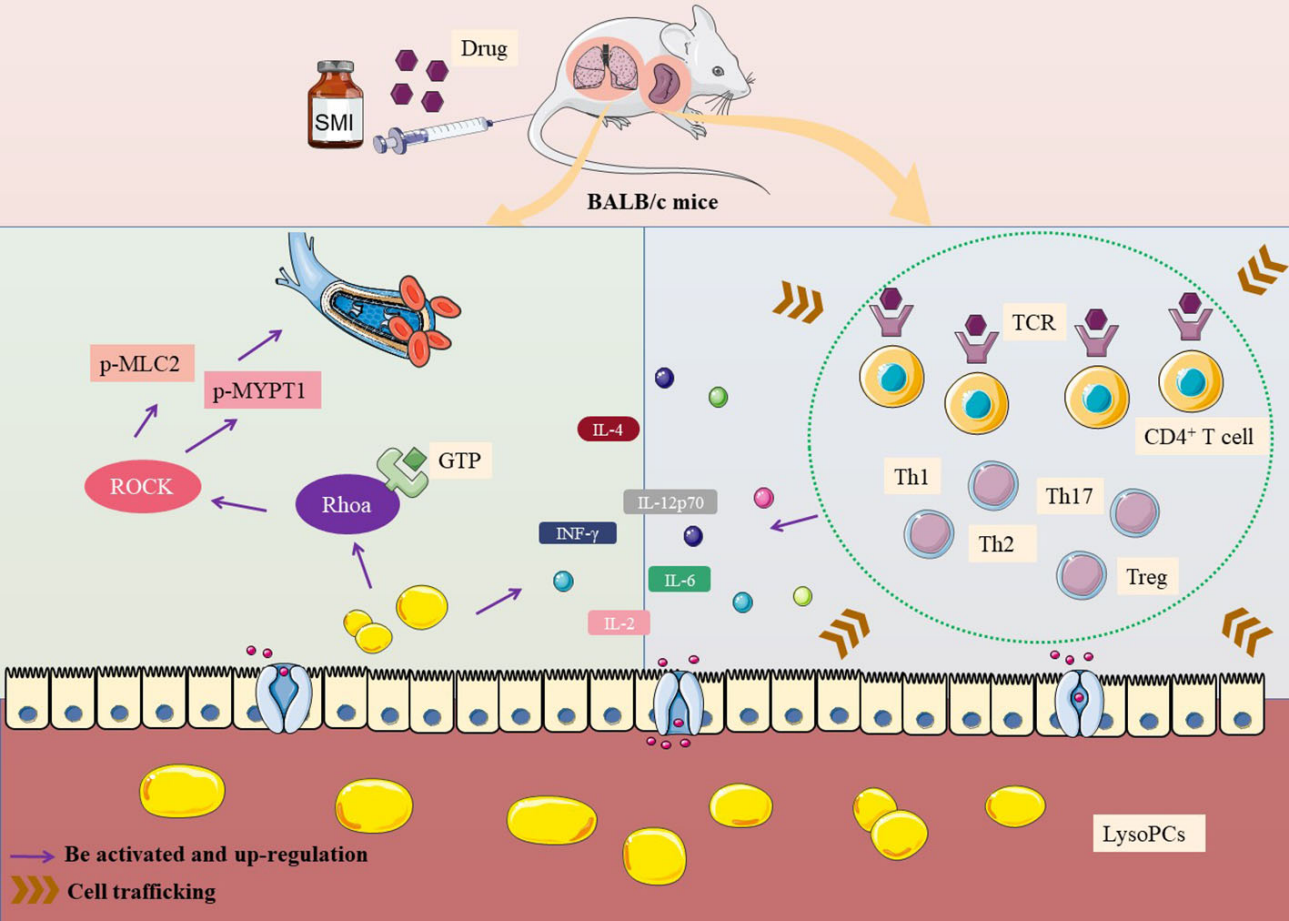
Mechanism of thymus-derived T cell-mediated immediate ADRs in BALB/c mice after injecting SMI.

The possible mechanism of thymus-derived T cell-mediated immediate ADRs in BALB/c mice after injecting SMI was shown in **Figure 5**.

## Conclusion

In this research, the mechanism of immediate ADRs induced by SMI was studied. By comparing the different *in vivo* responses generated by simultaneous injection of SMI in BALB/c mice and BALB/c nude mice, it was determined that the immediate ADRs were mediated by thymus-derived T cells. Moreover, SMI-induced immediate ADRs resulted in increased cytokine and vascular leakage levels in BALB/c mice. Furthermore, we found the metabolic profile of mice changed significantly after injecting SMI through further analysis of the metabolomics results. Lysolecithin metabolites, especially LysoPCs, played an important role in these immediate ADRs. At the same time, LysoPC (18:2(9Z,12Z)) had the potential to be a biomarker for SMI-induced immediate ADRs based on the correlation analysis of cytokine and metabolites. Finally, this study confirmed the activation of the RhoA/ROCK pathway, which was closely associated with increased levels of vascular leakage, cytokines and LysoPCs in BALB/c mice injected with SMI. The above results provided a new theoretical basis for the immediate ADRs induced by natural medicine injections.

## Data availability statement

The data presented in the study are deposited in the Nutstore repository at this link: https://www.jianguoyun.com/p/DRTgi90Q1Jy9Cxiwif0EIAA.

## Ethics statement

The animal study was reviewed and approved by Institutional Animal Care and Use Committee of the Institute of Chinese Materia Medica, China Academy of Chinese Medical Sciences.

## Author contributions

HZ, XW, and SJ developed the idea and designed the research. SJ, BS, YZha, JH, YZho, CP, LFW, NS. HW and XW performed the experiments. SJ analyzed the data. HZ, XW, BB and LNW revised the paper. All authors contributed to the article and approved the submitted version.
