# Supplementary material for: The immediate adverse drug reactions induced by ShenMai Injection are mediated by thymus-derived T cells and associated with RhoA/ROCK signaling pathway
Source: Front Immunol. 2023 Mar 21;14:1135701. doi: 10.3389/fimmu.2023.1135701 (PMC10070857; doi:10.3389/fimmu.2023.1135701)
Supplement: Supplementary file 2 [file Table_2.docx]

**Reagents**

Rabbit anti-p-MLC2 (Thr18/Ser19) (3674), rabbit anti-MLC2 (3672), rabbit anti-p-MYPT1 (Thr 696) (5163), rabbit anti-MYPT1 (2634), and rabbit anti-RhoA (67B9) (2117) were purchased from Cell Signaling Technology. Rabbit polyclonal antibody against glyceraldehyde-3-phosphate dehydrogenase (GAPDH) was purchased from Santa Cruz Biotechnology. Activated RhoA pull down assay kit was obtained from Cytoskeleton. Methanol, formic acid, and acetonitrile were obtained from Thermo Fisher Scientific.

ADRs induced by SMI
